# Assessing GPT-4’s Performance in Delivering Medical Advice: Comparative Analysis With Human Experts

**DOI:** 10.2196/51282

**Published:** 2024-07-08

**Authors:** Eunbeen Jo, Sanghoun Song, Jong-Ho Kim, Subin Lim, Ju Hyeon Kim, Jung-Joon Cha, Young-Min Kim, Hyung Joon Joo

**Affiliations:** 1 Department of Medical Informatics, Korea University College of Medicine, Seoul, Republic of Korea; 2 Department of Linguistics, Korea University, Seoul, Republic of Korea; 3 Korea University Research Institute for Medical Bigdata Science, Korea University, Seoul, Republic of Korea; 4 Department of Cardiology, Cardiovascular Center, Korea University College of Medicine, Seoul, Republic of Korea; 5 Division of Cardiology, Department of Internal Medicine, Korea University Anam Hospital, Seoul, Republic of Korea; 6 School of Interdisciplinary Industrial Studies, Hanyang University, Seoul, Republic of Korea

**Keywords:** GPT-4, medical advice, ChatGPT, cardiology, cardiologist, heart, advice, recommendation, recommendations, linguistic, linguistics, artificial intelligence, NLP, natural language processing, chatbot, chatbots, conversational agent, conversational agents, response, responses

## Abstract

**Background:**

Accurate medical advice is paramount in ensuring optimal patient care, and misinformation can lead to misguided decisions with potentially detrimental health outcomes. The emergence of large language models (LLMs) such as OpenAI’s GPT-4 has spurred interest in their potential health care applications, particularly in automated medical consultation. Yet, rigorous investigations comparing their performance to human experts remain sparse.

**Objective:**

This study aims to compare the medical accuracy of GPT-4 with human experts in providing medical advice using real-world user-generated queries, with a specific focus on cardiology. It also sought to analyze the performance of GPT-4 and human experts in specific question categories, including drug or medication information and preliminary diagnoses.

**Methods:**

We collected 251 pairs of cardiology-specific questions from general users and answers from human experts via an internet portal. GPT-4 was tasked with generating responses to the same questions. Three independent cardiologists (SL, JHK, and JJC) evaluated the answers provided by both human experts and GPT-4. Using a computer interface, each evaluator compared the pairs and determined which answer was superior, and they quantitatively measured the clarity and complexity of the questions as well as the accuracy and appropriateness of the responses, applying a 3-tiered grading scale (low, medium, and high). Furthermore, a linguistic analysis was conducted to compare the length and vocabulary diversity of the responses using word count and type-token ratio.

**Results:**

GPT-4 and human experts displayed comparable efficacy in medical accuracy (“GPT-4 is better” at 132/251, 52.6% vs “Human expert is better” at 119/251, 47.4%). In accuracy level categorization, humans had more high-accuracy responses than GPT-4 (50/237, 21.1% vs 30/238, 12.6%) but also a greater proportion of low-accuracy responses (11/237, 4.6% vs 1/238, 0.4%; *P*=.001). GPT-4 responses were generally longer and used a less diverse vocabulary than those of human experts, potentially enhancing their comprehensibility for general users (sentence count: mean 10.9, SD 4.2 vs mean 5.9, SD 3.7; *P*<.001; type-token ratio: mean 0.69, SD 0.07 vs mean 0.79, SD 0.09; *P*<.001). Nevertheless, human experts outperformed GPT-4 in specific question categories, notably those related to drug or medication information and preliminary diagnoses. These findings highlight the limitations of GPT-4 in providing advice based on clinical experience.

**Conclusions:**

GPT-4 has shown promising potential in automated medical consultation, with comparable medical accuracy to human experts. However, challenges remain particularly in the realm of nuanced clinical judgment. Future improvements in LLMs may require the integration of specific clinical reasoning pathways and regulatory oversight for safe use. Further research is needed to understand the full potential of LLMs across various medical specialties and conditions.

## Introduction

As a large language model (LLM), the GPT developed by OpenAI generates human-like text [1-3], distinguishing it from other specialized deep learning models that are limited to solving specific problems within predetermined domains [4]. In the medical field, GPT has the potential to augment medical education [5], provide clinical decision support [6], and enhance public health initiatives [7]. An impressive achievement of GPT-3.5 is its success in meeting the passing threshold for the United States Medical Licensing Examination [8], demonstrating its ability to offer medical advice comparable to that of trained professionals [9]. The latest iteration, GPT-4 [10,11], is anticipated to exhibit advancements in processing complex medical language, formulating patient care suggestions, and making preliminary diagnostic predictions, which inspires cautious optimism for its future applications in the medical domain [12].

Cardiovascular diseases are a leading cause of death worldwide, highlighting the critical need for precise and reliable information in this domain [13]. During the initial stages of the SARS-CoV-2 pandemic, overstated claims about the cardiovascular implications of the virus potentially escalated public unease and undermined trust in empirical findings [14]. The distribution of speculative or inaccurate information would have had a detrimental effect on the pandemic response strategies. It is paramount to emphasize that inaccuracies or misconceptions in cardiological advice can lead to severe consequences. Hence, there is a pressing need for rigorous validation of all sources of information, whether derived from human experts or advanced computational models such as GPT-4.

Moreover, the generation of “hallucinatory” or erroneous responses by GPT raises concerns about nonmedical expert users unintentionally accepting incorrect information as valid [15,16]. Consequently, proposals for regulatory oversight of LLMs have emerged, including the establishment of a new regulatory category specifically addressing LLM-related challenges and risks [4]. Therefore, it is crucial to develop auditing procedures capable of capturing the intricacies of LLM-associated risks, necessitating a balanced evaluation of the potential benefits and risks inherent in LLMs [17,18]. To delve deeper into this matter, this study applied real-world health consultations from general users to human experts through an internet portal, using the most recent iteration of this technology, GPT-4. The responses provided by both human experts and GPT-4 were subsequently evaluated by a panel of 3 independent cardiologists to gain a nuanced understanding of the potential benefits and risks associated with GPT-4.

## Methods

### Data Collection

**Figure 1.**
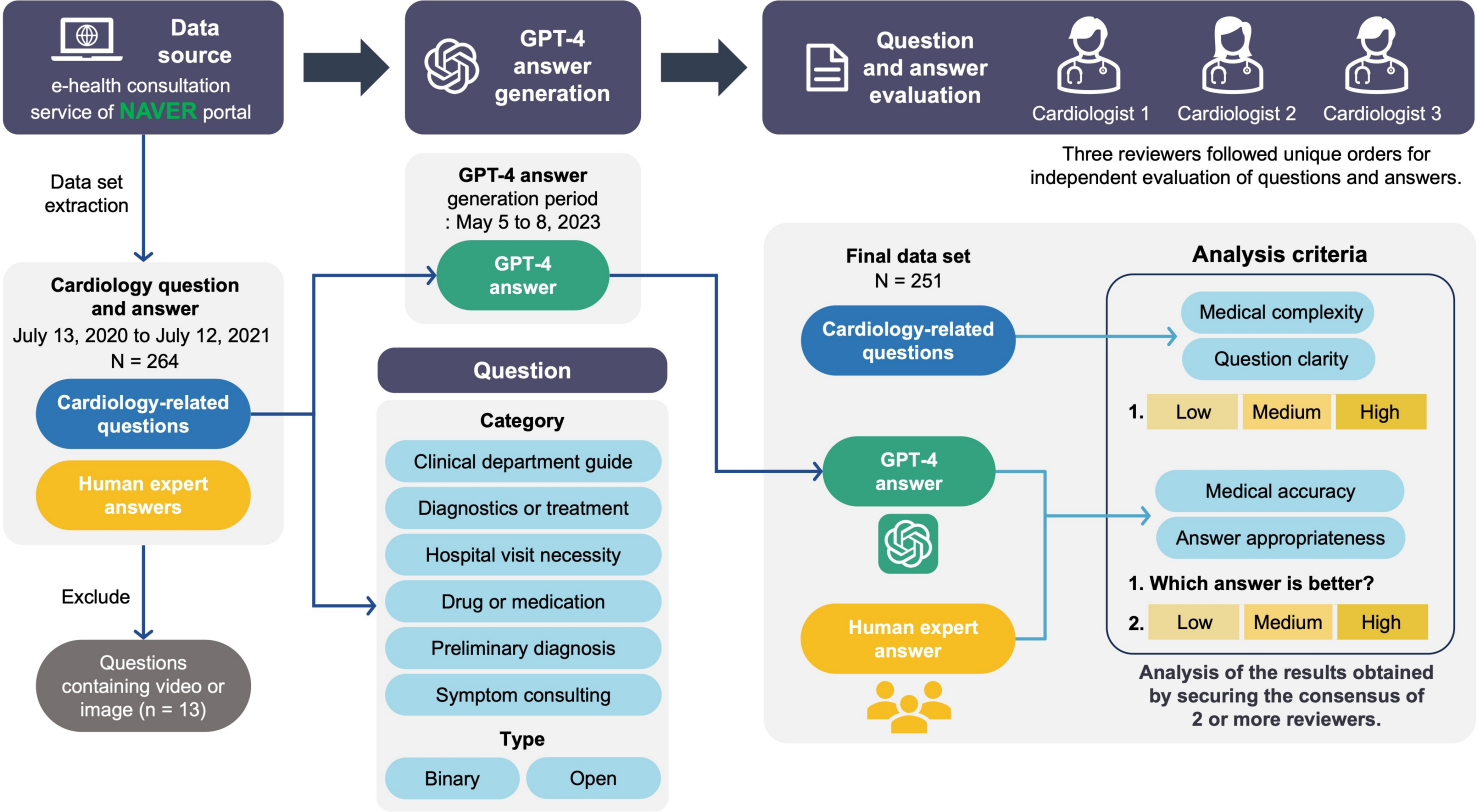
Study design and evaluation process. A data set consisting of 251 cardiology-specific question-answer pairs was collected from the NAVER portal over a 1-year period, from July 13, 2020, to July 12, 2021. A licensed medical professional is the person who answered the portal user’s question. The questions covered 6 domain categories and included both binary and open-ended types. From May 5 to 8, these questions were inputted into GPT-4 to generate the corresponding GPT-4 responses. Following that, a panel of 3 cardiologists reviewed and evaluated the questions along with the answers provided by human experts and GPT-4. The evaluation criteria focused on assessing the complexity and clarity of the questions as well as the accuracy and appropriateness of the responses from both human experts and GPT-4.

Figure 1 illustrates the study design. We collected question-and-answer data related to cardiology from the Korean search portal NAVER, focusing on 264 cases. NAVER is Korea’s largest search engine, and its web-based questions and answers forums, called “Jisik-In,” have previously been used in medical research [19,20]. The data set covered the period from July 13, 2020, to July 12, 2021, and included medical inquiries posed by portal users and the corresponding responses provided by human experts. These experts are doctors who have graduated from a college of medicine or medical school, passed the Korean Medical Licensing Examination, and hold legal accreditations as certified specialists in their respective medical fields from the Ministry of Health and Welfare. They are not restricted by character limits when answering users’ questions on the portal site. The questions were categorized into 2 types: binary and open-ended. Further, 6 distinct categories were defined based on the questions’ intent. All collected data were in Korean text form. To ensure the analysis was focused on sufficiently detailed and substantive exchanges, we specifically selected questions that contained more than 100 characters according to the Korean alphabet and answers provided by human experts that comprised at least 200 characters. This approach was aimed at filtering out overly simple queries and ensuring that the responses were elaborate enough for a thorough comparison. Additionally, to maintain a consistent and fair comparison basis between the capabilities of GPT-4 and human experts, we excluded 13 cases from the total data set that contained multimedia content such as videos or images. Finally, 251 cases were selected for the study after applying these criteria.

### GPT Answer Generation

Answers to the collected questions were generated using OpenAI’s GPT-4 model, released on March 14, 2023 [10]. From May 5 to 8, 2023, a total of 3 researchers used this model via the OpenAI website to generate GPT-4 answers. The total data set of questions to be entered into the GPT-4 was distributed to the 3 researchers in the form of a spreadsheet. Each original Korean question was directly fed into the GPT-4 prompt without any supplementary input. The researchers saved the generated answer in a spreadsheet. Each question input was done in a new session by clicking the “New chat” button.

### Question and Answer Evaluation

Once the data were randomly shuffled, answers from both GPT-4 and human experts were anonymized and labeled as answer 1 and answer 2, respectively, ensuring the 3 independent cardiologist reviewers were blinded to the source of each response. Each of these reviewers is a board-certified physician in internal medicine and has undergone more than 4 years of fellow training in cardiology subspecialty. A panel of 3 cardiologists assessed the question set along with the anonymized answers. The evaluation was conducted using a computer interface. Each evaluator assessed the clarity and complexity of the questions as well as the accuracy and appropriateness of the answers. To quantitatively measure these aspects, a 3-tiered grading scale (low, medium, and high) was used (Multimedia Appendix 1). Additionally, each evaluator determined which answer (the GPT-4’s answer or the human expert’s answer) showed superior accuracy and appropriateness in relation to the question posed.

To further elucidate, the Kendall *W* concordance analysis revealed the following coefficient values indicating the level of agreement among the evaluators: 0.44 for the appropriateness of the human expert answers, 0.40 for the appropriateness of the GPT-4 answers, 0.43 for the medical accuracy of the human expert answers, and 0.40 for the medical accuracy of the GPT answers. Moreover, when making a binary choice determining the superiority of appropriateness between the human expert and GPT-4 answers, the coefficient was 0.42, and for determining the superiority of medical accuracy between the two, it was 0.45. These values, falling in the range of 0.40-0.60, denote a moderate agreement, showcasing a significant level of reliability in our study findings.

### Ethical Considerations

This research project was approved by the institutional review board of Korea University Anam Hospital (IRB 2023AN0280). The research was conducted in accordance with the Helsinki Declaration. Informed consent was obtained from all 3 participating cardiologists.

### Linguistic Analysis

The Korean Sentence Separator 4.5.1 was used to segment the text into individual sentences. For text tokenization, the Korean medical bidirectional encoder representations from the transformer model, which was specifically designed for Korean medical text analysis, was used [21]. To evaluate lexical diversity, the type-token ratio (TTR) was computed for each set of responses [22,23]. The TTR, which represents the ratio of unique words to the total number of words in a text, was determined after the responses were tokenized [22,23].

### Statistical Analysis

To discern statistically significant differences across categorical outcomes, we used the chi-square test or Fisher exact test as appropriate, depending on the expected frequencies within the categories. For continuous variables, comparison across groups was conducted using either the parametric unpaired 2-tailed *t* test or the nonparametric Mann-Whitney test, based on the distribution of the data. Interrater agreement among the 3 cardiologist evaluators was quantitatively assessed using the Kendall *W* concordance analysis. The association between the complexity and clarity of questions and the quality of responses was investigated using the Spearman rank correlation coefficient. All statistical analyses were conducted using SAS 9.4 (SAS Institute Inc) and R program (version 3.6.1; R Foundation for Statistical Computing).

## Results

Both the number of words and sentences per answer were significantly higher for GPT-4 answers than for human expert answers (word count: mean 190, SD 75.2 for GPT-4 vs mean 139, SD 95.6 for humans; *P*<.001 and sentence count: mean 10.9, SD 4.2 for GPT-4 vs mean 5.9, SD 3.7 for humans; *P*<.001; Table 1). The GPT-4 answers exhibited lower lexical diversity, as measured by the TTR, compared to the answers provided by human experts. This suggests that GPT-4 answers may be perceived as more comprehensible and similar to human conversations rather than written text (TTR: mean 0.69, SD 0.07 for GPT-4 vs mean 0.79, SD 0.09 for humans; *P*<.001).

**Table 1.** Linguistic difference between GPT-4 and human expert answers.

Characteristics	GPT-4, mean (SD)	Human, mean (SD)	*P* value
Word count per answer	190 (75.2)	139 (95.6)	<.001
Sentence count per answer	10.9 (4.2)	5.9 (3.7)	<.001
Type-token ratio	0.69 (0.07)	0.79 (0.09)	<.001

**Figure 2.**
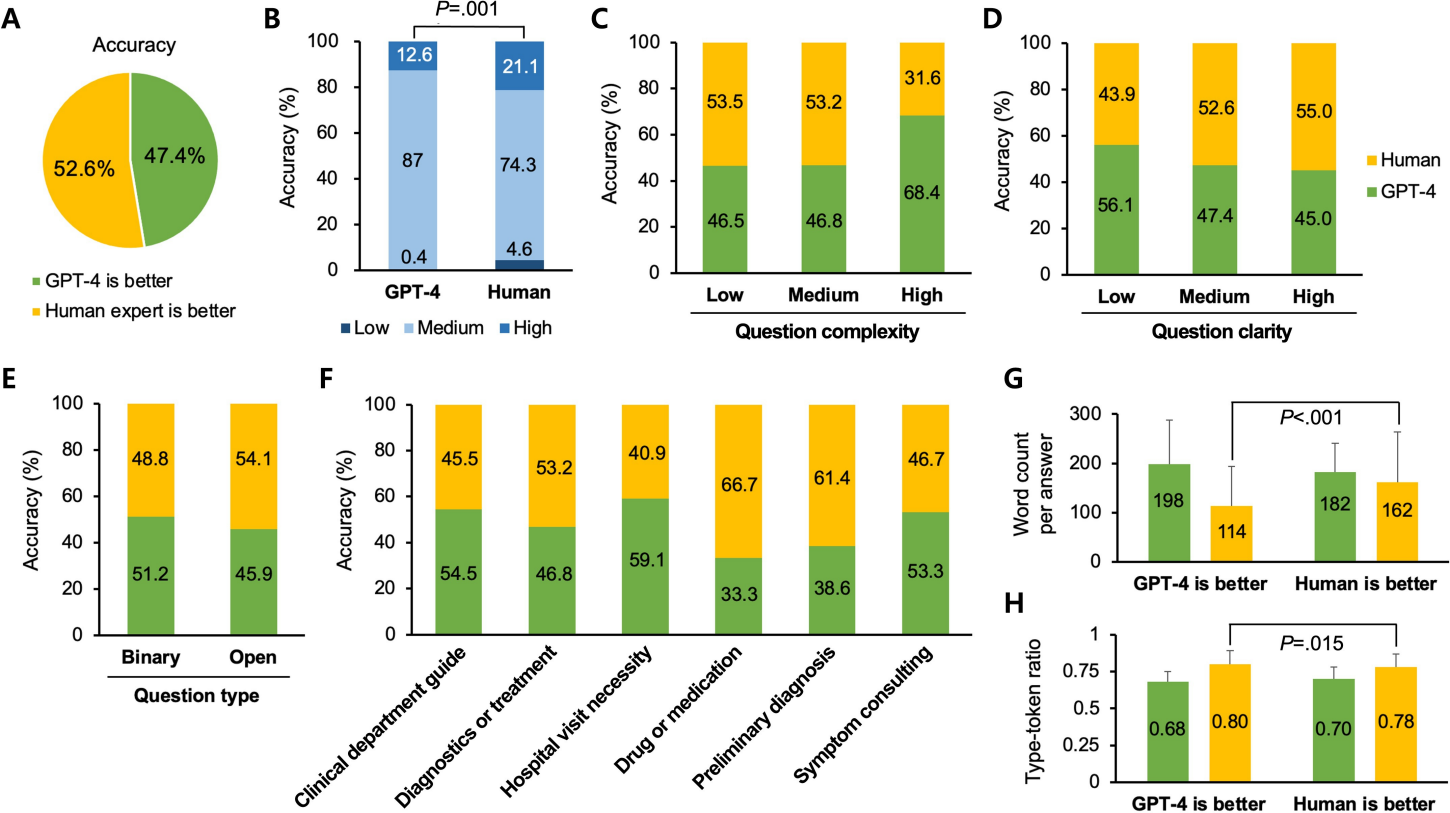
Medical accuracy between GPT-4 and human expert answers. (A) Survey results indicating preference for GPT-4 and human expert answers based on perceived medical accuracy. (B) Analysis of perceived medical accuracy, categorized as low, medium, and high for both GPT-4 and human expert answers. (C and D) Relationship between question complexity or clarity and the perceived medical accuracy of GPT-4 and human expert answers. (E) Comparison of variations in perceived medical accuracy between GPT-4 and human expert answers, depending on question type. (F) Comparison of perceived medical accuracy between GPT-4 and human expert answers across different categories of question intent. (G and H) Comparison of word count per answer and type-token ratio between human expert and GPT-4 answers when evaluated for medical accuracy.

Figure 2 presents an analysis of the medical accuracy between GPT-4 and human expert answers. When cardiologists were asked to evaluate which answers were more medically accurate, the responses slightly favored the human expert answers (132/251, 52.6% vs 119/251, 47.4%; *P*=.41; Figure 2A). Dividing medical accuracy into low, medium, and high levels, a significant proportion of human expert answers were ranked as highly accurate compared to GPT-4 (50/237, 21.1% vs 30/238, 12.6%; *P*<.001; Figure 2B). However, the rate of low accuracy was also higher for the human expert answers (11/237, 4.6% vs 1/238, 0.4%; *P*=.007). This counterintuitive observation underscores the potential of LLMs to bridge gaps in human work in real-world scenarios.

**Figure 3.**
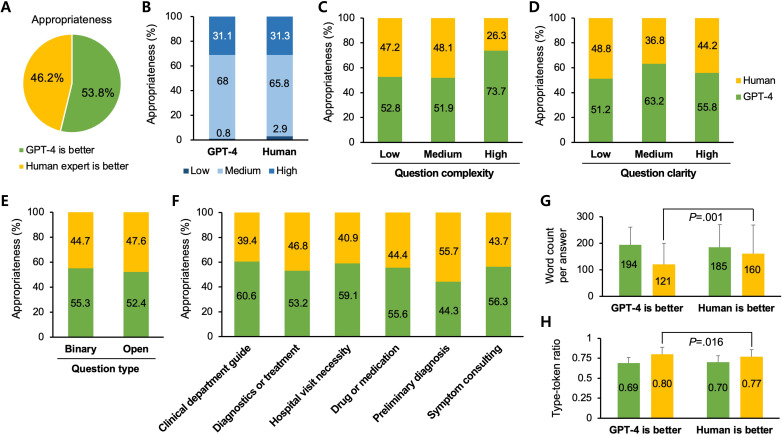
Answer appropriateness between GPT-4 and human expert answers. (A) Survey results indicating preference for GPT-4 and human expert responses based on perceived answer appropriateness. (B) Analysis of perceived answer appropriateness, categorized as low, medium, and high for both GPT-4 and human expert answers. (C and D) Relationship between question complexity or clarity and the perceived answer appropriateness of GPT-4 and human expert answers. (E) Comparison of variations in perceived answer appropriateness between GPT-4 and human expert answers depending on question type. (F) Comparison of perceived answer appropriateness between GPT-4 and human expert answers across different categories of question intent. (G and H) Comparison of word count per answer and type-token ratio between human expert and GPT-4 answers when evaluated for appropriateness.

In terms of question complexity and ambiguity, GPT-4 demonstrates an advantage. The more complex and ambiguous the question, the higher the medical accuracy of GPT-4’s answers. Conversely, human experts excel in dealing with simpler and clearer questions, although without statistically significant differences (*P*=.19; Figure 2C and *P*=.30; Figures2D, 3C,  3D). The difference in medical accuracy between human and GPT-4 answers remained below 10% across different question types (*P*=.39; Figure 2E).

Interestingly, when analyzing question categories based on the intent, numerical differences were observed, but without statistical significance (*P*=.20; Figure 2F). Human experts outperformed GPT-4 in responding to questions related to drugs or medications and preliminary diagnoses, scoring higher than GPT-4 (drug or medication: 12/18, 66.7% vs 6/18, 33.3% and preliminary diagnosis: 43/70, 61.4% vs 27/70, 38.6%). Conversely, GPT-4 surpassed human experts in addressing queries regarding the necessity of hospital visits and guidance for clinical departments (hospital visit necessity: 9/22, 40.9% vs 13/22, 59.1% and clinical department guidance: 15/33, 45.5% vs 18/33, 54.5%).

In the linguistic analysis, when the medical accuracy of a human expert’s answer exceeded that of GPT-4, the human expert’s answers typically had a higher word count and lower TTR compared to cases where GPT-4’s answers were deemed more medically accurate (word count per answer: mean 162, SD 102.6 vs mean 114, SD 80.3; *P*<.001; Figure 2G and TTR: mean 0.78, SD 0.09 vs mean 0.80, SD 0.09; *P*=.02; Figure 2H). This implies that the more the response resembles a real conversation—longer and easier—the higher the perceived medical accuracy according to cardiology experts. This observation indicates a potential area for quality control in human expert responses and highlights the consistent performance of GPT-4 in terms of response length and lexical variation.

Next, a comparative analysis between GPT-4 and human expert answers was conducted in terms of answer appropriateness (Figure 3). When assessing whether GPT-4 or human expert answers were more appropriate for the posed questions, GPT-4 was rated as superior (GPT-4: 135/251, 53.8% vs humans: 116/251, 46.2%; *P*=.23; Figure 3A). Similar to the medical accuracy analysis, when categorizing appropriateness into low, medium, and high, both GPT-4 and human expert answers showed a comparable distribution across these segments (*P*=.26; Figure 3B). Notably, mirroring the findings from the medical accuracy analysis, the frequency of answers deemed to have low appropriateness was numerically higher for human experts (7/240, 2.9% vs 2/241, 0.8%; *P*=.03), suggesting the possibility of human shortcomings. The investigations related to question complexity, clarity, and type displayed numerical trends similar to those observed in the medical accuracy analysis, although no statistical differences were observed (*P*=.20; *P*=.60; and *P*=.66; Figure 3C-E). The analysis based on question intent showed no significant statistical discrepancies between the proportions of cases where human expert answers were deemed more appropriate and those where GPT-4 answers were considered more appropriate. Interestingly, GPT-4 was rated as more appropriate than human experts in all other categories, except for the question category of preliminary diagnosis (*P*=.58; Figure 3F). When human expert answers were considered more appropriate than those of GPT-4, the corresponding answers had a higher word count and lower TTR compared to cases where GPT-4 answers were deemed more appropriate (word count per answer: mean 121, SD 79.3 vs mean 160, SD 108.1; *P*=.001; Figure 3G and TTR: mean 0.80, SD 0.09 vs mean 0.77, SD 0.09; *P*=.02; Figure 3H). Similar to medical accuracy, these findings suggest that longer responses resembling genuine conversations are evaluated as more appropriate.

**Figure 4.**
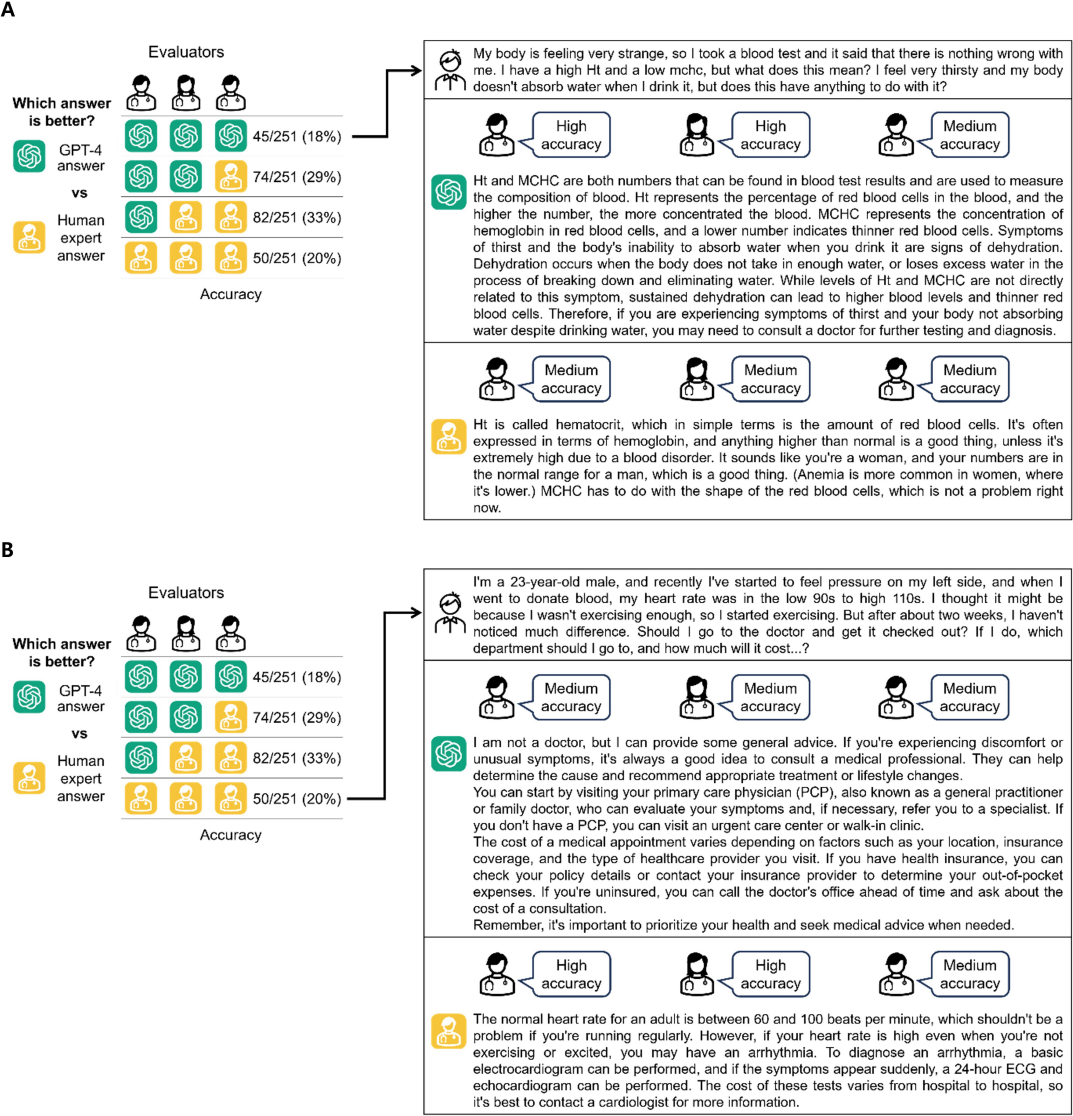
Evaluation result and representative cases comparing medical accuracy between GPT-4 and human expert answers. (A) A case where the GPT-4 answer received superior medical accuracy ratings from all 3 evaluators. (B) A case where a human expert received superior medical accuracy ratings from all 3 evaluators.

**Figure 5.**
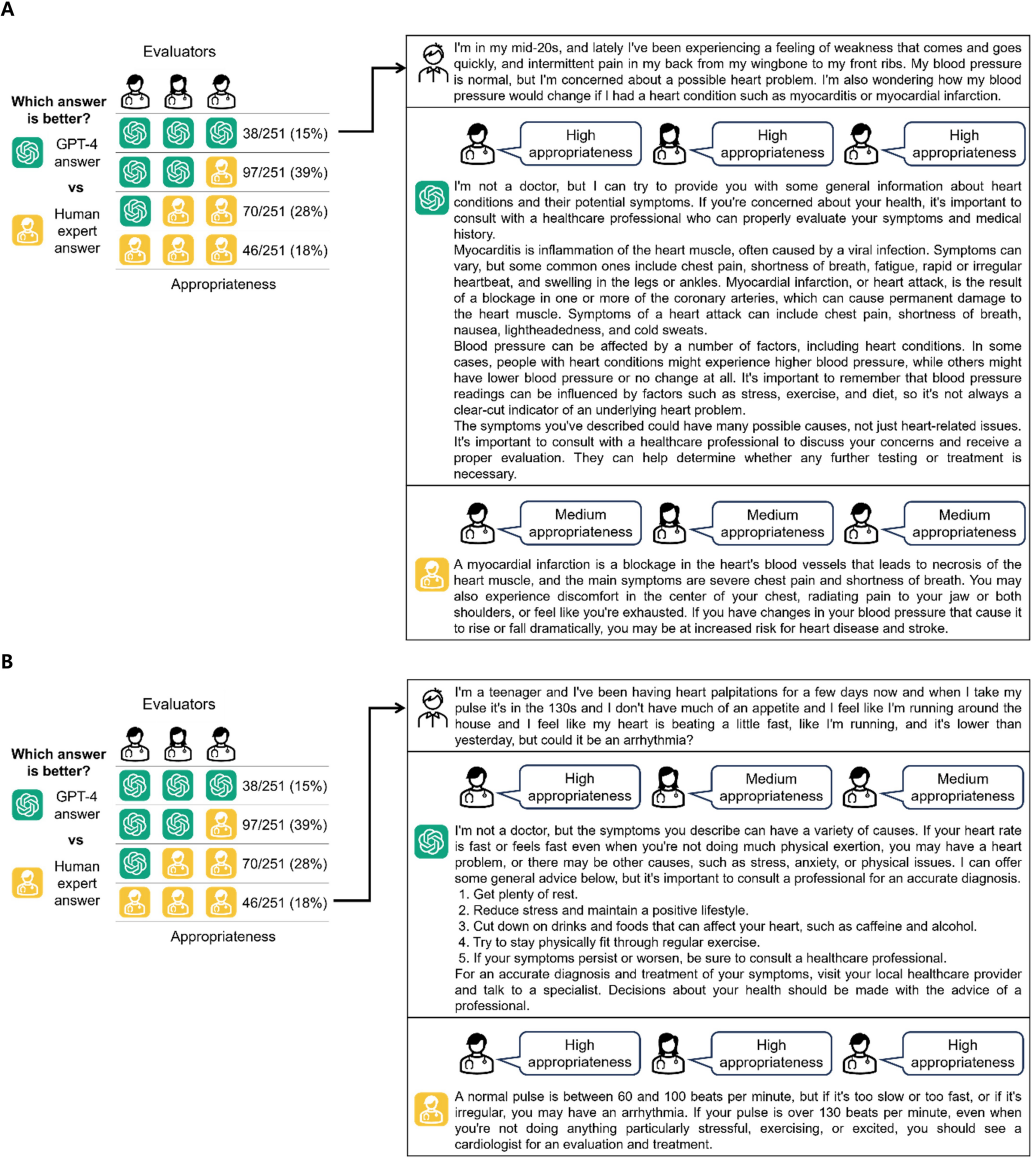
Evaluation result and representative cases comparing answer appropriateness between GPT-4 and human expert answers. (A) A case where the GPT-4 answer received superior appropriateness ratings from all 3 evaluators. (B) A case where a human expert answer received superior appropriateness ratings from all 3 evaluators.

For the 251 questions assessed, all 3 independent cardiologists rated the GPT-4 answers as superior in 18% (45/251) of cases in terms of medical accuracy. In an additional 29% (74/251) of the cases, the majority (2 of 3) of cardiologists endorsed the GPT-4 answers. Conversely, human expert answers were unanimously considered more accurate in 20% (50/251) of cases, with the majority of cardiologists agreeing with human experts in 33% (82/251) of cases (Figure 4). In terms of answer appropriateness, all 3 cardiologists agreed that the GPT-4 answers were superior in 15% (38/251) of cases. The majority of cardiologists found GPT-4 answers to be more appropriate in another 39% (97/251) of cases. Human experts, however, received unanimous approval for the appropriateness of their answers in 18% (70/251) of cases and majority approval in an additional 28% (46/251; Figure 5). These figures highlight the noteworthy performance of GPT-4 from a medical standpoint. Examining illustrative cases, GPT-4 stands out for delivering medical information resembling the content of medical textbooks and dictionaries. Additionally, GPT-4 demonstrates strength in thoroughly addressing every user’s question, leaving no queries unanswered. In contrast, human experts leverage their advantage in providing heuristic information informed by their clinical experience, especially when questions require elements of clinical judgment.

## Discussion

### Principal Findings

This research uniquely implemented real-world health consultations involving general users and human experts, comparing the answers provided by human experts and GPT-4. Three independent cardiologists appraised the answers to discern the potential advantages and disadvantages of using GPT-4 in the medical advice domain. This study demonstrated comparable levels of medical accuracy between GPT-4 and human experts. Notably, human expert answers had a higher proportion of answers classified as having low medical accuracy compared to those from GPT-4.

Another significant finding suggests the benefits of articulating medical advice in a conversational style, which positively impacts medical accuracy and relevance to queries. This style proved effective in responding to all questionnaire requests, leading to higher answer ratings and demonstrating the potential of GPT-4 in providing medical advice. Notably, GPT-4’s answers consistently displayed appropriate length and lexical variation compared to those of human experts. The findings of this study underscore the potential of GPT-4 in medical education, particularly in enhancing the learning experience through its ability to simulate conversational medical advice with accuracy comparable to human experts. Integrating GPT-4 into educational frameworks could offer an innovative approach to medical education, facilitating adaptive learning and preparing students for the digital evolution in health care. This suggests a promising avenue for future research and application in the field of medical education, highlighting the importance of incorporating advanced AI tools like GPT-4 to complement traditional educational methods.

### Comparison to Prior Work

An important consideration is the linguistic scope of our findings. This study was conducted in Korean, which naturally raises questions about its generalizability to other languages. Recent studies and OpenAI’s own documentation suggest that GPT-4’s performance in non-English languages, including medical contexts, has improved compared to previous versions [11,24,25]. Takagi et al [24] compared the performance of GPT-3.5 and GPT-4 using 254 questions from the Japanese Medical Licensing Examination, revealing that GPT-4 exhibited a 29.1% improvement over GPT-3.5. They highlighted that GPT-4’s enhanced non-English language processing capabilities were instrumental in its ability to pass the medical licensing examination. In addition, Wang et al [25] conducted a study comparing the performance of GPT-3.5 and GPT-4 on English and Chinese data sets for the Chinese Medical Licensing Examination, showing a significant improvement in accuracy for Chinese compared to English. This study showed that the medical advice provided by GPT-4 was comparable in medical accuracy to that provided by human experts. Based on previous research and the findings of this study, it has been found that GPT-4 can effectively process specialized medical information in various non-English languages, including Korean. This indicates its potential for use in patient education and the dissemination of medical knowledge.

### Strengths and Limitations

Despite its strengths, GPT-4’s capability to provide advice based on clinical experience differs notably from that of human experts. Furthermore, quantitative analysis revealed potential discrepancies between GPT-4 and human expert responses, depending on the intent of the question. Numerous studies are currently underway to identify appropriate regulatory measures for the use of LLMs [4]. The findings of this investigation are anticipated to facilitate subsequent research aimed at identifying tasks in the medical field that GPT-4 excels in. This, in turn, could expedite the development of technology to enhance the quality of medical services and promote public health.

This study has several limitations to consider. First, its focus on cardiology might limit the generalizability of the results to other medical specialties. Second, the sample size for the answer evaluation, which consisted of only 3 cardiologists, could have been larger for a more robust analysis. Furthermore, since the evaluations were conducted solely by cardiologists, there is potential for reporting bias where certain aspects of the answers might be overemphasized or underrepresented. Inclusion of professionals from other domains could have provided a broader assessment. Future studies should aim to involve larger sample sizes and encompass a wider range of medical specialties. Moreover, integrating patients’ perspectives could offer further insights into the acceptability and perceived utility of artificial intelligence–powered medical advice.

### Conclusions

In conclusion, this study revealed the promising capabilities of GPT-4 in providing medically accurate and appropriate responses comparable to human experts. The additional benefits of GPT-4 include consistent proficiency in maintaining appropriate response length and lexical variation. However, GPT-4 showed some disadvantages in providing advice based on clinical experience as well as variation in its performance depending on question intent. Despite these challenges, this study suggests that LLMs such as GPT-4 hold significant potential in augmenting medical education, providing medical advice.

## Supplementary material

10.2196/51282 Multimedia Appendix 1 Standards for evaluating medical questions and answers.

## Abbreviations

LLM: large language model
TTR: type-token ratio

## References

[1] Alberts IL, Mercolli L, Pyka T (2023). Large language models (LLM) and Chatgpt: what will the impact on nuclear medicine be?. Eur J Nucl Med Mol Imaging.

[2] Nath S, Marie A, Ellershaw S, Korot E, Keane PA (2022). New meaning for NLP: the trials and tribulations of natural language processing with GPT-3 in ophthalmology. Br J Ophthalmol.

[3] Floridi L, Chiriatti M (2020). GPT-3: its nature, scope, limits, and consequences. Minds Mach.

[4] Meskó B, Topol EJ (2023). The imperative for regulatory oversight of large language models (or generative AI) in healthcare. NPJ Digit Med.

[5] Abd-Alrazaq A, AlSaad R, Alhuwail D (2023). Large language models in medical education: opportunities, challenges, and future directions. JMIR Med Educ.

[6] Lu Y, Wu H, Qi S, Cheng K (2023). Artificial intelligence in intensive care medicine: toward a ChatGPT/GPT-4 way?. Ann Biomed Eng.

[7] Biswas SS (2023). Role of ChatGPT in public health. Ann Biomed Eng.

[8] Kung TH, Cheatham M, Medenilla A (2023). Performance of ChatGPT on USMLE: potential for AI-assisted medical education using large language models. PLOS Digit Health.

[9] Cheng K, Sun Z, He Y, Gu S, Wu H (2023). The potential impact of ChatGPT/GPT-4 on surgery: will it topple the profession of surgeons?. Int J Surg.

[10] GPT-4 is OpenAI’s most advanced system, producing safer and more useful responses. OpenAI.

[11] OpenAI (2024). GPT-4 technical report. arXiv.

[12] Goktas P, Karakaya G, Kalyoncu AF, Damadoglu E (2023). Artificial intelligence chatbots in allergy and immunology practice: where have we been and where are we going?. J Allergy Clin Immunol Pract.

[13] Mensah GA, Roth GA, Fuster V (2019). The global burden of cardiovascular diseases and risk factors: 2020 and beyond. J Am Coll Cardiol.

[14] Frangogiannis NG (2020). The significance of COVID-19-associated myocardial injury: how overinterpretation of scientific findings can fuel media sensationalism and spread misinformation. Eur Heart J.

[15] Lee P, Bubeck S, Petro J (2023). Benefits, limits, and risks of GPT-4 as an AI chatbot for medicine. N Engl J Med.

[16] Duffourc M, Gerke S (2023). Generative AI in health care and liability risks for physicians and safety concerns for patients. JAMA.

[17] Singhal K, Azizi S, Tu T (2023). Large language models encode clinical knowledge. Nature.

[18] Reddy S (2023). Evaluating large language models for use in healthcare: a framework for translational value assessment. Inform Med Unlocked.

[19] Jo W, Kim Y, Seo M, Lee N, Park J (2022). Online information analysis on pancreatic cancer in Korea using structural topic model. Sci Rep.

[20] Jo W, Lee J, Park J, Kim Y (2020). Online information exchange and anxiety spread in the early stage of the novel coronavirus (COVID-19) outbreak in South Korea: structural topic model and network analysis. J Med Internet Res.

[21] Kim Y, Kim JH, Lee JM (2022). A pre-trained BERT for Korean medical natural language processing. Sci Rep.

[22] Das A, Verma RM (2020). Can machines tell stories? A comparative study of deep neural language models and metrics. IEEE Access.

[23] Miao J, Zhang Y, Jiang N (2023). Towards unifying pre-trained language models for semantic text exchange. Wireless Netw.

[24] Takagi S, Watari T, Erabi A, Sakaguchi K (2023). Performance of GPT-3.5 and GPT-4 on the Japanese Medical Licensing Examination: comparison study. JMIR Med Educ.

[25] Wang H, Wu W, Dou Z, He L, Yang L (2023). Performance and exploration of ChatGPT in medical examination, records and education in Chinese: pave the way for medical AI. Int J Med Inform.

