# Supplementary material for: Assessing GPT-4’s Performance in Delivering Medical Advice: Comparative Analysis With Human Experts
Source: JMIR Med Educ. 2024 Jul 8;10:e51282. doi: 10.2196/51282 (PMC11250047; doi:10.2196/51282)
Supplement: Multimedia Appendix 1 [file mededu-v10-e51282-s001.docx]

| **Standards** | | **Definition** |
| --- | --- | --- |
| Question complexity | High | The question invokes sophisticated medical paradigms, conditions, or treatments, could harbor multiple valid resolutions, contingent on a myriad of factors, or situates within a sphere of considerable scholarly contention or progressive research. |
|  | Medium | The question necessitates extensive comprehension of medical theories or afflictions, might entail multiple plausible answers, hinging on the context, or could instigate certain degree of scholarly dissension. |
|  | Low | The question entails foundational medical knowledge, yields an unambiguous and uncomplicated solution, and is not anticipated to arouse disparate scholarly opinions. |
| Question clarity | High | The question is impeccably formulated and readily grasped, absent of any ambiguity or perplexity in its conveyance. |
|  | Medium | The query is somewhat lucid but might mandate further elucidation or rewording for comprehensive reader understanding. |
|  | Low | The query is burdensome to interpret, filled with ambiguous wording or unclear terminology that impedes the reader's discernment of the principal subject. |
| Medical accuracy | High | The answer aligns with current medical standards and practices, offers pertinent advice or data. It is devoid of medical inaccuracies and provides information with both depth and breadth, as substantiated by current evidence-based medical knowledge. |
|  | Medium | The answer incorporates some germane details but might harbor minor inaccuracies or outdated advice, or it may lack holistic comprehensiveness. |
|  | Low | The answer is incorrect or disseminates misleading data, contravening medical norms or practices. |
| Answer appropriateness | High | The answer is directly pertinent to the query, offering beneficial intelligence or guidance. |
|  | Medium | The answer is somewhat related but may encompass some unrelated or redundant data. |
|  | Low | The answer is not germane or deviates markedly from the context of the query. |
